# Influence of Arthroplasty Type, Comorbidities, and Fracture Status on Outcomes After Shoulder Replacement: Analysis of 664,545 Cases

**DOI:** 10.3390/healthcare14040427

**Published:** 2026-02-08

**Authors:** Assil Mahamid, Miri Elgabsi, Muhammad Khatib, Hamza Murad, Feras Qawasmi, Eitan Lavon, Ali Yassin, Mustafa Yassin

**Affiliations:** 1 Department of Orthopedics, Hasharon Hospital, Rabin Medical Center, Affiliated to Tel Aviv University, Tel Aviv 6997801, Israel; miriv42@gmail.com (M.E.); muhammad.kh@hotmail.de (M.K.); ferasport@yahoo.com (F.Q.);; 2 Gray Faculty of Medical & Health Sciences, Tel Aviv University, Tel Aviv 6997801, Israel; 3 Department of General Surgery, Hillel Yaffe Medical Center, Hadera 3820302, Israel; 4 Management Department, Hasharon Hospital at Clalit Health Services, Rabin Medical Center, Affiliated to Tel Aviv University, Tel Aviv 6139001, Israel; 5 Department of Orthopedic Surgery, Hillel Yaffe Medical Center, Hadera 3820302, Israel

**Keywords:** anatomic total shoulder arthroplasty, reverse total shoulder arthroplasty, shoulder hemiarthroplasty, National inpatient sample, postoperative complications

## Abstract

**Background**: Shoulder arthroplasty is performed for various etiologies, including osteoarthritis, proximal humerus fractures (PHFs), and rotator cuff tears. While previous studies have focused on outcomes based on implant choice, less is known about the independent effects of surgery type, comorbidities, and fracture status on postoperative outcomes. This study evaluates their influence on length of stay (LOS), in-hospital mortality, and postoperative complications. **Methods**: A retrospective cohort analysis of 664,545 patients undergoing anatomic total shoulder arthroplasty (ATSA), reverse total shoulder arthroplasty (RTSA), or hemiarthroplasty (HA) was conducted. Multivariable Poisson and logistic regression models assessed predictors of LOS, mortality, and complications. **Results**: Among 132,909 patients, 63.3% underwent RTSA, 31.3% underwent ATSA, and 5.4% underwent HA. Mean hospitalization was longest for HA (2.56 days) and RTSA (1.82 days) compared to ATSA (1.39 days; *p* < 0.001). Poisson regression confirmed that RTSA increased LOS by 24.1% versus ATSA (IRR = 1.24, *p* < 0.001), while HA had the highest LOS (IRR = 1.58, *p* < 0.001). Postoperative complications were observed in 8.37% of ATSA, 13.81% of RTSA, and 17.81% of HA cases (overall ~12.3%). Compared with ATSA, RTSA increased the odds of complications (OR = 1.48, *p* < 0.001), while HA presented the greatest complication risk (OR = 1.51, *p* < 0.001). Among proximal humerus fracture (PHF) patients (9.9% of the cohort), 84.7% underwent RTSA. PHF independently increased LOS (IRR = 1.61, *p* < 0.001), mortality (OR = 1.62, *p* = 0.051), and complications (OR = 2.33, *p* < 0.001). **Conclusions**: RTSA is associated with longer hospitalization and higher complication rates, while PHF worsens LOS, mortality, and complication risk.

## 1. Introduction

Shoulder hemiarthroplasty (HA) has been consistently associated with higher 30-day complication rates—including adverse events, mortality, sepsis, transfusion, and prolonged hospitalization—when compared with anatomic total shoulder arthroplasty (ATSA) for osteoarthritis, as demonstrated in recent propensity-matched analyses [1]. Reverse total shoulder arthroplasty (RTSA) also exhibits elevated early complication rates, particularly instability, scapular fracture, infection, and all-cause complications, relative to an ATSA. Nonetheless, long-term outcomes appear comparable, with revision rates at 5 years being similar between RTSA and ATSA. Notably, some large database studies have reported higher overall complication and revision rates for aTSA, likely reflecting the influence of extended follow-up periods in those cohorts [2,3,4].

Perioperative complications such as mortality, pneumonia, venous thromboembolism, and transfusion are reported more frequently in RTSA compared with TSA, even after adjustment for comorbid conditions [5]. Earlier literature suggested comparable short-term complication profiles between HA and TSA; however, more contemporary evidence indicates a significantly higher risk associated with HA [1,6,7]. Patient-specific factors including age, comorbidity burden, and surgical indication (e.g., fracture or inflammatory arthritis) exert a profound influence on complication risk across all arthroplasty types, in some cases outweighing the effect of the procedure itself [4,7,8,9,10]. For example, both inflammatory arthritis and traumatic indications are consistently associated with higher complication rates irrespective of implant type [8,9].

Surgical indication remains a critical determinant of in-hospital postoperative outcomes. Patients undergoing arthroplasty for inflammatory arthritis experience disproportionately higher rates of both medical and surgical complications—including deep wound infection, implant loosening, mechanical failure, pulmonary embolism, and acute blood loss anemia—compared with those treated for osteoarthritis [9]. Likewise, proximal humeral fractures and other traumatic indications are associated with elevated short-term risks of venous thromboembolism and surgical site infection compared with degenerative conditions [7,8,11]. Specifically, fracture nonunion, avascular necrosis, and acute fractures have all been linked to increased in-hospital infection risk relative to primary osteoarthritis [11].

RTSA is more commonly performed in elderly patients and in complex scenarios such as fracture or rotator cuff arthropathy. These patient populations are particularly vulnerable, demonstrating higher rates of perioperative complications—including mortality, pneumonia, venous thromboembolism, and transfusion—even after controlling for comorbidities and demographic variables [3,5,12]. Collectively, the evidence suggests that in-hospital complication risk is more strongly determined by patient comorbidities, age, and surgical indication than by the specific arthroplasty procedure itself. Certain indications, particularly trauma, fracture, and inflammatory arthritis, independently predict higher rates of adverse outcomes [6,7,8,9,11].

Although prior studies have reported higher complication rates following reverse total shoulder arthroplasty and hemiarthroplasty compared with anatomic total shoulder arthroplasty, these associations have often been examined in isolation or within restricted patient subsets. Less attention has been given to the relative contribution of surgical indication, particularly fracture status, and patient comorbidity burden in shaping in-hospital outcomes at a national level. Accordingly, the objective of this study was not solely to compare arthroplasty types, but to provide an integrated, population-based assessment of how procedure selection, fracture-related indications, and patient complexity interact to influence short-term outcomes in contemporary shoulder arthroplasty practice.

Against this backdrop, the present large-scale retrospective analysis of 664,545 patients undergoing shoulder arthroplasty aimed to delineate the independent effects of surgical procedure and fracture status on postoperative outcomes. The findings highlight those patients undergoing RTSA or HA experienced longer hospitalizations and a greater likelihood of complications compared with those treated with ATSA.

## 2. Materials and Methods

### 2.1. Data Source

The National Inpatient Sample (NIS), developed by the Agency for Healthcare Research and Quality (AHRQ) as part of the Healthcare Cost and Utilization Project (HCUP), was used as the data source for this study. The NIS is a discharge-level database derived from a stratified probability sample of approximately 20% of inpatient hospitalizations from HCUP-affiliated hospitals, representing roughly seven million unweighted admissions annually. Application of HCUP-provided discharge weights (DISCWT) enables extrapolation to nationally representative estimates. The present analysis utilized NIS data from 1 January 2016 through 31 December 2021, corresponding to the most recent data available at the time of study conduct. Each NIS record represents a single inpatient hospitalization; when weighted, each unweighted discharge corresponds, on average, to approximately five hospitalizations at the national level, although the exact weight varies by year and sampling stratum. Detailed descriptions of the NIS sampling design and weighting methodology are publicly available in the HCUP NIS Design and Weighting documentation.

### 2.2. Cohort Definition and Selection Criteria

The National Inpatient Sample (NIS) database was queried for the years 2016–2021 to identify adult patients (aged > 18 years) who underwent shoulder replacement surgery. Surgical procedures were classified using ICD-10 procedure codes into three categories: reverse total shoulder arthroplasty (RTSA), anatomic total shoulder arthroplasty (ATSA), and hemiarthroplasty (HA) (see Table 1 for procedure codes). A total of 132,909 unweighted hospital discharges were identified, corresponding to a nationally representative estimate of 664,545 patients after application of discharge weights. Unless otherwise specified, all analyses were conducted on the full cohort of shoulder arthroplasty cases, including reverse, anatomic, and hemiarthroplasty procedures.

Because the NIS is a publicly available, fully de-identified database, this study does not constitute human subjects research; therefore, in accordance with Healthcare Cost and Utilization Project (HCUP) guidelines and U.S. federal regulations (45 CFR §46), institutional review board approval and informed consent were not required. More on that is available at: https://hcup-us.ahrq.gov/nisoverview.jsp (accessed on 1 February 2025).

### 2.3. Outcome Variables (End Points)

The primary objective of this study was to evaluate the impact of surgical procedure type on clinical outcomes and complications following shoulder replacement surgery. These complications included venous thromboembolism (deep vein thrombosis [DVT] and pulmonary embolism [PE]), surgical site infection, cardiac complications (myocardial infarction, cardiac arrest), respiratory complications (pneumonia, respiratory failure), and acute renal failure. Continuous outcome variables, LOS and hospital charges, were dichotomized. Patients with a LOS above the 75th percentile of inpatient duration were classified as having “prolonged LOS.” Similarly, those with hospital charges above the 75th percentile were classified as having “high-end hospital charges.

### 2.4. Exposure Variables & Statistical Analysis

The patient-level characteristics analyzed included age, sex, race, primary payer, elective status and comorbidities. Comorbidities of interest included heart disease (hypertension and congestive heart failure), chronic obstructive pulmonary disease, chronic renal disease, type 2 diabetes mellitus, alcohol abuse, Parkinson’s disease, Alzheimer’s disease, mental disorders, dyslipidemia, chronic anemia, and obstructive sleep apnea (Table 1). Hospital-level characteristics included hospital bed size, location (urban or rural), academic status (teaching vs. non-teaching), and geographical region.

Demographic, clinical, and hospital characteristics of patients undergoing shoulder replacement surgery were compared across surgery types using Pearson’s χ^2^ test for categorical variables. For continuous variables, normality tests were performed, and since they did not follow a normal distribution, the Kruskal–Wallis test was used. Descriptive statistics were presented as counts and percentages for categorical variables, while continuous variables were summarized as mean or median with interquartile range (IQR), as applicable. Hospital charges were rounded to the nearest whole number. Given the large sample size, statistical significance should be interpreted in conjunction with effect size and clinical relevance, as small but statistically significant associations may not translate into meaningful clinical impact.

Missing data were most prevalent in race (3.74%), primary payer (0.10%), and hospitalization cost (0.70%), while lower proportions were observed in in-hospital mortality (0.02%), sex (0.02%), age (0.00%), and length of stay (0.00%). Assuming a missing at random (MAR) mechanism, multiple imputation by chained equations (MICE) was applied. Continuous variables were imputed using Bayesian Ridge regression, binary variables with logistic regression, and categorical variables with most frequent imputation, ensuring biologically plausible values. Comparative analysis confirmed no significant differences between imputed and original datasets, validating the imputation process. The final imputed dataset was used for all analyses without introducing substantial bias.

The analysis used multivariable logistic and Poisson regression models to identify predictors of complications and hospital outcomes among patients undergoing shoulder replacement surgery, with surgery type as the independent variable. Multicollinearity was assessed, and no significant collinearity was detected. This study aimed to identify factors influencing complications and hospital outcomes and assess surgery type’s impact on prolonged length of stay, in-hospital mortality, and postoperative complications. These outcomes were selected based on univariate analyses. Given the prespecified, hypothesis-driven nature of the analyses, formal adjustment for multiple comparisons was not applied; results were interpreted in the context of effect sizes, confidence intervals, and clinical relevance. All analyses were conducted in Python 3.10 using the Pandas library and appropriate statistical packages, with statistical significance set at *p* ≤ 0.001.

AI tools were used solely to improve the clarity, grammar, and style of the manuscript in English. These tools were not employed for data analysis, interpretation, or content generation.

## 3. Results

### 3.1. Patient Demographics, Comorbidities, and Hospital Characteristics by Shoulder Replacement Surgery Type

The study included a total of 664,545 weighted patients who underwent shoulder replacement surgery; among these, 207,895 underwent ATSA, 420,680 underwent RTSA, and 35,970 underwent HA. The overall mean age was 69.6 years, with RTSA patients being the oldest (71.3 years) compared to ATSA (66.8 years) and HA (65.0 years). Females accounted for 56.5% of the cohort, with the highest proportion observed in the RTSA group (60.2%). Most patients were White (89.1%) and predominantly insured by Medicare (70.7%). While a range of comorbidity burdens was noted—with 35.1% of patients having 0–1 comorbidity and 15.5% having four or more—common conditions included diabetes, hypertension, and dyslipidemia. In terms of hospital characteristics, approximately one-third of patients were treated in small hospitals, one-quarter in medium-sized hospitals, and nearly 42% in large hospitals, with the majority admitted to urban teaching facilities. All differences across procedure groups were statistically significant (*p* < 0.001). Table 2 provides a comprehensive summary of these findings.

**Figure 1:**
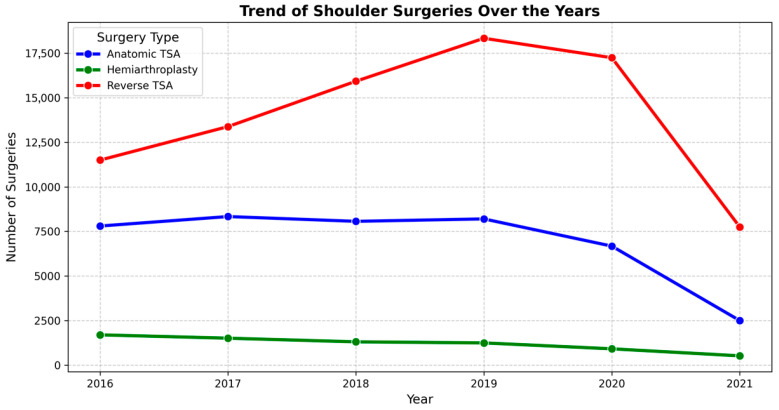
Illustrates the annual trends in shoulder replacement surgeries from 2016 to 2021. RTSA remained the most commonly performed procedure throughout the study period. A steady increase in all surgery types was observed until 2019, followed by a decline, particularly in 2020.

**Figure 2:**
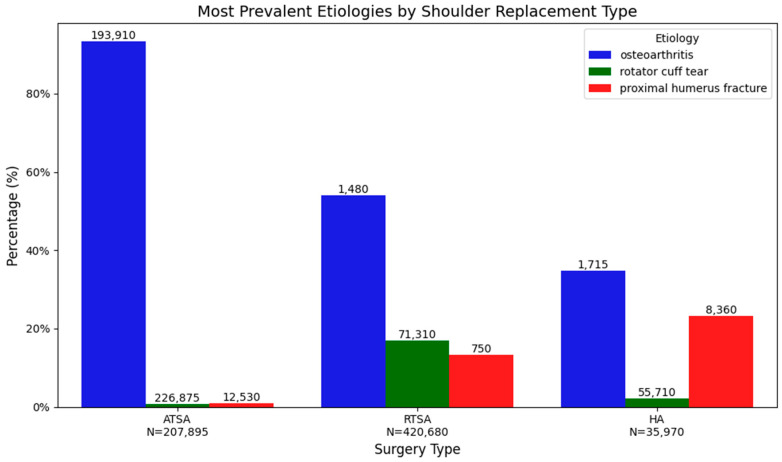
The most prevalent etiologies by Shoulder Replacement Type.

Figure 1 demonstrates the shoulder surgery volumes increased steadily from 2016 to a peak around 2019–2020, driven primarily by a marked rise in reverse total shoulder arthroplasty, while ATSA remained relatively stable and hemiarthroplasty gradually declined. In 2021, all procedure types showed a sharp drop, with the most pronounced reduction seen in RTSA and ATSA, likely reflecting the impact of the COVID-19 pandemic. Osteoarthritis was the most common etiology (65.2% overall), occurring in 93.3% of ATSA cases, 53.9% of RTSA cases, and 34.9% of HA cases. Rotator cuff tear and proximal humerus fracture were more frequent in RTSA (16.96% and 13.24%, respectively) and HA (2.09% and 23.24%) than in ATSA (0.71% and 0.83%). All intergroup differences were statistically significant (*p* < 0.001). Surgical etiology is summarized in Table 3, and the three most prevalent complications by surgery are displayed in Figure 2.

### 3.2. Univariate Analysis of Complications and Hospital Outcomes by Shoulder Replacement Surgery Type

Our analysis identified significant associations between surgery type and multiple factors, including prolonged length of stay, gender, race, primary payer, comorbidities and postoperative complications (*p* < 0.05). Hospital characteristics were initially significant but lost significance after Bonferroni correction. Cramér’s V indicated mostly weak associations. The strongest were surgery etiology (0.346), prolonged length of stay (0.153), and primary payer (0.126). Postoperative complications, classified as present or absent, showed a moderate association with surgery type (Cramér’s V = 0.086). Comorbidities, categorized into four groups (0–1, 2, 3, and ≥4 conditions), were also significantly associated with surgery type (Cramér’s V = 0.059).

Complications were observed in 17,395 (8.37%) of ATSA patients, 58,115 (13.81%) of RTSA patients, and 6405 (17.81%) of HA patients. Compared with ATSA, RTSA patients had significantly higher odds of complications (OR 1.48, 95% CI 1.43–1.54), as detailed in Table 4. Length of hospital stay and charges varied significantly by surgery type (*p* < 0.001). HA patients had the highest rate of prolonged stays (30.1%), followed by reverse (18.3%) and anatomic (8.6%). Similarly, RTSA patients were most likely to incur high-end costs (28.4%), compared to partial (24.5%) and anatomic (17.6%).

In-hospital mortality was low across all groups (0.03% ATSA, 0.06% RTSA, 0.21% HA; OR 1.10). HA had the lowest elective surgery rate (80.61%) compared to RTSA (92.65%) and ATSA (97.02%) (OR 0.72, 95% CI 0.69–0.75).

### 3.3. Comparison of Hospitalization Outcomes

Total hospitalization charges and length of stay differed significantly among surgical groups (*p* < 0.001). The median charges were $57,767 (IQR: $42,525–$79,946) for ATSA, $68,523 (IQR: $50,393–$96,464) for RTSA, and $59,521 (IQR: $40,948–$90,196) for HA, with an overall median of $64,684 (IQR: $47,239–$90,864). Similarly, length of stay differed significantly (*p* < 0.001); while both ATSA and RTSA patients had a median LOS of 1 day (IQR: 1–2), HA patients had a longer median stay of 2 days (IQR: 1–3) [Table 5].

### 3.4. Impact of Proximal Humerus Fractures on Surgical Outcomes in Shoulder Arthroplasty

Fracture patients had distinct clinical and demographic characteristics. RTSA was the primary procedure (84.7%), while ATSA was rare (2.6%), whereas non-fracture patients underwent RTSA less often (60.9%) and ATSA more frequently (34.4%). Fracture patients were older (72.2 vs. 69.3 years), and women were more commonly affected (80.0% vs. 53.9%). Postoperative outcomes were significantly worse for fracture patients. Complications were nearly three times more common (28.1% vs. 10.6%), in-hospital mortality was five times higher (0.22% vs. 0.04%), and hospitalization lasted more than twice as long (3.19 vs. 1.57 days). Additionally, 42.1% of fracture patients incurred high-end hospital charges compared to 22.9% of non-fracture patients.

Multivariable regression confirmed that proximal humerus fractures independently contributed to worse outcomes. Fractures increased the incidence of prolonged hospitalization by 61.0% (IRR = 1.61, *p* < 0.001) and raised the odds of in-hospital mortality by 62.4% (OR = 1.62, *p* = 0.051). Patients with fractures were also 133.1% more likely to develop postoperative complications (OR = 2.33, *p* < 0.001), with older age, female sex, and higher comorbidity burden further elevating risk.

### 3.5. Multivariable Analysis Results

Poisson regression confirmed that surgery type was a significant predictor of hospital length of stay (LOS). Compared to ATSA, RTSA extended hospitalization by 24.1% (IRR = 1.24, *p* < 0.001), while HA resulted in the longest LOS, increasing hospital stay by 58% (IRR = 1.58, *p* < 0.001). A greater comorbidity burden was significantly associated with a progressively longer hospital stay. Compared to patients with 0–1 comorbidities, those with two conditions had a 7.0% increase in LOS (IRR = 1.07, *p* < 0.001), three comorbidities increased LOS by 15.8% (IRR = 1.16, *p* < 0.001), and those with four or more comorbidities experienced the longest hospital stay, with a 28.9% increase in LOS (IRR = 1.29, *p* < 0.001).

Logistic regression revealed that surgery type was not an independent predictor of in-hospital mortality (OR = 1.46, *p* = 0.118). However, prolonged LOS (OR = 2.59, *p* < 0.001), postoperative complications (OR = 39.2, *p* < 0.001), and high-end hospital charges (OR = 3.15, *p* < 0.001) were the strongest predictors of mortality. For postoperative complications, RTSA patients had a 22.3% higher odds of developing complications compared to ATSA (OR = 1.22, *p* < 0.001), while HA had the highest complication risk (OR = 1.51, *p* < 0.001). Increased comorbidity burden further raised complication risk, with OR = 1.59 (*p* < 0.001) for two comorbidities and OR = 1.91 (*p* < 0.001) for three or more. Prolonged LOS was the most significant predictor, increasing complication risk by 4.4-fold (OR = 4.40, *p* < 0.001).

**Figure 3:**
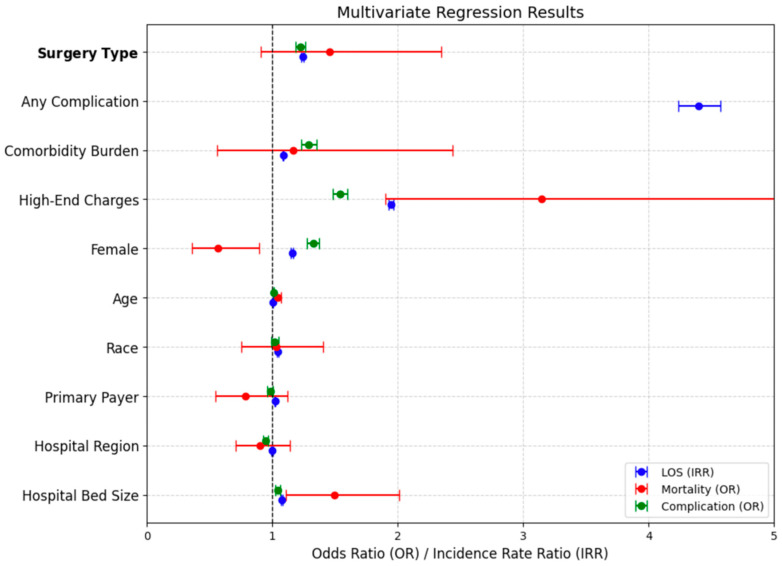
Illustrates Multivariate Regression of Predictors for LOS, Mortality, and Postoperative Complications.

The multivariate regression results are visualized in Figure 3. The plot displays odds ratios (ORs) and incidence rate ratios (IRRs) for key predictors of hospital length of stay (LOS), in-hospital mortality, and postoperative complications.

## 4. Discussion

This large-scale retrospective analysis of 664,545 patients provides a comprehensive evaluation of outcomes following shoulder arthroplasty, emphasizing the influence of surgical procedure and proximal humerus fracture status. RTSA and HA were consistently associated with longer hospital stays and higher complication rates compared with ATSA. These differences are partly explained by patient demographics and surgical indications, as RTSA and HA were more often performed in older patients with complex etiologies such as rotator cuff arthropathy and proximal humerus fracture. Importantly, proximal humerus fracture emerged as a powerful, independent predictor of adverse outcomes, conferring markedly higher risks of postoperative complications, prolonged hospitalization, and in-hospital mortality. These findings highlight the critical role of procedure selection, surgical indication, and patient-specific factors in risk stratification and perioperative planning.

While our findings confirm previously reported associations between arthroplasty type and postoperative complications, the principal contribution of this study lies in the comparative contextualization of these risks within a large, contemporary national cohort. Specifically, our results demonstrate that fracture status and overall patient complexity exert a greater influence on in-hospital outcomes than implant choice alone. This observation underscores the importance of surgical indication and baseline patient factors in perioperative risk stratification and challenges interpretations that attribute adverse outcomes primarily to arthroplasty type.

Evidence from large database studies and meta-analyses consistently demonstrates that RTSA is associated with increased perioperative complication rates and longer length of stay compared with ATSA, particularly in patients with osteoarthritis or related degenerative conditions [2,5,13,14,15,16]. Reported complications include infection, instability, periprosthetic fracture, pneumonia, venous thromboembolism, and transfusion, with higher risks persisting even after adjustment for age and comorbidity burden. Similarly, HA is associated with higher complication rates and longer hospitalization than ATSA in patients with glenohumeral osteoarthritis and intact rotator cuffs. Propensity-matched and meta-analytic data further suggest elevated risks of death, sepsis, transfusion, revision, and overall adverse events following HA compared with ATSA [1,17]. While select subgroups such as patients with avascular necrosis may demonstrate lower complication and revision rates with HA relative to ATSA, these findings are not generalizable to broader osteoarthritis populations [18].

Given the exceptionally large sample size, even very small differences reached statistical significance. Accordingly, some associations—such as odds ratios close to unity (e.g., OR ≈ 1.10)—may be statistically significant yet of limited clinical relevance. In contrast, effect sizes of greater magnitude, particularly those observed for proximal humerus fractures (e.g., OR > 2 for complications and IRR > 1.5 for prolonged length of stay), likely represent clinically meaningful risk differentials. These findings underscore the importance of interpreting statistical significance in the context of effect size and baseline risk, especially in large database studies.

Proximal humerus fracture represents a particularly high-risk indication for shoulder arthroplasty. Patients with fractures are typically older, present with more comorbidities, and are often acutely medically compromised, which increases perioperative risk and resource utilization [7,13,19,20]. The acute fracture setting is associated with greater surgical complexity, longer operative times, higher transfusion requirements, and an increased likelihood of discharge to extended care facilities, all contributing to prolonged hospitalization and greater complication rates [7,13]. Compared with degenerative indications, fracture patients face substantially elevated risks of infection, hematoma, mechanical failure, revision surgery, and mortality, with odds of short-term complications reported to be more than threefold higher [7,20,21]. These outcomes reflect not only the technical challenges of surgery in the acute trauma setting but also the frailty and physiologic vulnerability of the patient population.

RTSA is also consistently associated with higher hospitalization costs compared with other forms of shoulder arthroplasty, largely driven by elevated implant costs, greater complication rates, longer operative times, and prolonged hospitalization. The prosthesis itself represents a substantial cost driver, with RTSA implants incurring significantly higher supply expenses than ATSA or HA [22,23,24]. Moreover, RTSA is more often performed in older, comorbid patients presenting with complex indications such as proximal humerus fractures, further contributing to increased resource utilization [5,25,26]. Complications such as transfusion, pneumonia, venous thromboembolism, and myocardial infarction amplify hospitalization costs through extended recovery, additional interventions, and increased rates of discharge to skilled nursing facilities [5,13,26,27]. Collectively, these findings underscore that both clinical and economic outcomes following shoulder arthroplasty are shaped not only by procedure type but also by patient factors and surgical indication.

This study leverages the NIS, the largest publicly available all-payer inpatient database in the United States, enabling robust, nationally representative estimates across a large and diverse patient population. The use of weighted discharge data ensures generalizability to real-world clinical practice. Rigorous methodology—including strict inclusion criteria, comprehensive comorbidity adjustment, multiple imputation for missing data, and application of multivariable logistic and Poisson regression models—strengthens the validity of the findings. By stratifying outcomes by surgical procedure type and fracture status, this study provides nuanced insights into risk profiles that extend beyond implant design to highlight the impact of patient comorbidities and surgical indication. The large sample size further allows for the detection of clinically meaningful associations, even among rare outcomes such as in-hospital mortality.

Several limitations warrant consideration. First, as a retrospective analysis of an administrative database, this study is inherently dependent on the accuracy and completeness of ICD-10 coding, which may introduce misclassification bias and preclude assessment of procedure-specific technical details. Reliance on administrative codes may result in misclassification of both surgical indications and postoperative complications, particularly with respect to fracture complexity, differentiation between acute and chronic pathology, and procedure-specific nuances. Such misclassification is likely non-differential and would be expected to bias effect estimates toward the null rather than generate spurious associations. Moreover, despite adjustment for a comprehensive set of demographic and comorbidity variables, residual confounding may persist due to unmeasured factors, including patient frailty, bone quality, injury severity, surgeon experience, hospital procedural volume, and perioperative care pathways. Consequently, the observed associations should be interpreted as correlational rather than causal. Second, the National Inpatient Sample lacks granular clinical information, such as implant characteristics, surgical techniques, surgeon-level variables, and postoperative functional outcomes, all of which may influence complication risk and longer-term prognosis. Third, although multiple imputation techniques were employed to address missing data, the possibility of residual bias related to incomplete data cannot be fully excluded. Fourth, outcomes were limited to the inpatient setting, precluding evaluation of long-term complications, reoperations, and functional recovery. Finally, while multivariable modeling was used to adjust for measured confounders, unmeasured variables and residual confounding may still influence the reported associations.

## 5. Conclusions

In conclusion, shoulder arthroplasty outcomes are strongly influenced by procedure type, fracture status, and patient comorbidities. RTSA and HA were linked to longer hospitalizations and higher complication rates than ATSA, while proximal humerus fractures independently predicted worse outcomes, including higher mortality. Patient factors had a greater impact than procedure choice, highlighting the importance of individualized risk assessment and perioperative optimization.

## Figures and Tables

**Table 1:** ICD-10 diagnostic and procedure codes used to define study variables.

Category	Subcategory	ICD10_Codes
Shoulder Replacement Surgery Type	Anatomic total shoulder replacement	0RRJ0JZ, 0RRK0JZ, 0RRJ0KZ, 0RRJ07Z 0RRK0KZ*, 0RRK07Z*
	Reverse total shoulder replacement	0RRJ00Z, 0RRK00Z
	Partial shoulder replacement	0RRJ0J7, 0RRK0J7, 0RRJ0J6, 0RRK0J6
Surgical Etiology	Osteoarthritis	M00*, M08*, M10*, M13*, M15*, M16*, M17*, M19*, M86*
	Rotator cuff tear	M66*, M67*, M75*, M79*
	Proximal humerus fracture	M97*, S42*, S52*
	Complication	T81*, T84*, T85*, T86*
	Arthropathies	G56*, M07*, M11*, M12*, M14*
	Avascular necrosis	M87*
	Deformity	M21*, M24*, Q74*, Q77*
	Inflammatory arthritis	M05*, M06*
	Malunion/nonunion	M80*, M81*, M84*, M85*
	Malignancy	C40*, C41A2*, C49*, C79*, C90*, D16*, D48*, Z85*
	Other	A15*, A41*, A52*, A69*, C34*, C41*, C49*, C50*, C71*, C7A*, C7B*, C83*, C85*, C90*, D16*, D48*, D57*, D62*, D64*, D66*, E03*, E11*, E13*, E22*, E27*, E53*, E75*, E78*, E83*, E86*, E87*, F03*, F10*, F11*, F17*, F29*, F32*, F33*, F39*, G20*, G25*, G30*, G40*, G47*, G54*, G56*, G71*, G89*, G90*, G92*, G93*, G97*, H83*, I10*, I11*, I12*, I13*, I16*, I21*, I25*, I26*, I44*, I48*, I50*, I63*, I67*, I69*, I72*, I95*, I97*, J15*, J18*, J43*, J44*, J45*, J47*, J69*, J95*, J96*, J98*, K21*, K25*, K29*, K52*, K55*, K56*, K92*, K94*, L03*, L40*, L51*, L94*, M07*, M08*, M10*, M11*, M14*, M16*, M17*, M18*, M1A*, M41*, M48*, M54*, M61*, M62*, M65*, M66*, M67*, M77*, M79*, M81*, M85*, M89*, M90*, M94*, M95*, M96*, N12*, N17*, N18*, N30*, N36*, N39*, N52*, N99*, Q74*, Q77*, R06*, R07*, R09*, R11*, R13*, R29*, R41*, R42*, R56*, R73*, S02*, S06*, S12*, S14*, S22*, S27*, S29*, S32*, S40*, S44*, S46*, S47*, S49*, S52*, S54*, S72*, S82*, S92*, T40*, T42*, T43*, T50*, T78*, T79*, T80*, T82*, T83*, T86*, T88*, U07*, W18*, W19*, X58*, Z01*, Z02*, Z03*, Z23*, Z42*, Z45*, Z47*, Z51*, Z68*, Z79*, Z85*, Z87*, Z89*, Z91*, Z96*
Comorbidity	Diabetes	E11-14*
	Hypertension	I10*
	Dyslipidemia	E78 *
	Obstructive Sleep Apnea	G473 *
	Anemia	D64 *
	Alcohol Abuse	F10 *
	Mental Disorders	F *
	Alzheimer’s Disease	G30 *
	Parkinson’s Disease	G20
	Chronic Kidney Disease	N18 *
	Chronic Obstructive Pulmonary Disease	J44 *
	Heart Failure	I50 *
Complications	Surgical Site Infection	T814 *
	Urinary Tract Infection	N39 *
	Pneumonia	J18 *, J15 *, J22
	Respiratory Failure	J96 *
	Cardiac Arrest	I46 *
	Myocardial Infarction	I20-24 *
	Acute Renal Failure	N17 *
	Embolism (DVT, PE)	I2602, I2609, I2692, I2699, I82401-I82429
	Acute Blood Loss	D62 *

The asterisk (*) in a code indicates a wildcard, meaning it includes all codes that begin with the specified characters followed by additional characters.

**Table 2:** Patient Demographics, Comorbidities, and Hospital Characteristics by Shoulder Replacement Surgery Type.

Characteristic	Overall (N = 664,545)	ATSA (n = 207,895)	RTSA (n = 420,680)	HA (n = 35,970)	*p*-Value
**Age, years (mean ± SD)**	69.57 ± 9.37	66.80 ± 9.25	71.33 ± 8.55	65.02 ± 13.11	<0.001
**Female, n (%)**	375,535 (56.5)	102,540 (49.3)	253,330 (60.2)	19,665 (54.7)	<0.001
**Race, n (%)**					<0.001
White	592,145 (89.1)	187,445 (90.2)	374,115 (88.9)	30,585 (85.0)	
Black	29,650 (4.5)	9215 (4.4)	18,130 (4.3)	2305 (6.4)	
Hispanic	26,320 (4.0)	6240 (3.0)	18,295 (4.4)	1785 (5.0)	
Other	16,430 (2.5)	4995 (2.4)	10,140 (2.4)	1295 (3.6)	
**Payer, n (%)**					<0.001
Medicare	470,085 (70.7)	127,945 (61.5)	321,035 (76.3)	21,105 (58.7)	
Medicaid	22,275 (3.4)	7960 (3.8)	11,670 (2.8)	2645 (7.4)	
Private	139,370 (21.0)	62,210 (29.9)	67,415 (16.0)	9745 (27.1)	
Other	32,815 (4.9)	9780 (4.7)	20,560 (4.9)	2475 (6.9)	
**Comorbidities Category, n (%)**					<0.001
0–1 Comorbidities	233,375 (35.1)	83,355 (40.09)	136,385 (32.42)	13,635 (37.91)	
2 Comorbidities	186,425 (28.05)	58,030 (27.91)	119,100 (28.31)	9295 (25.84)	
3 Comorbidities	141,505 (21.29)	40,030 (19.25)	94,235 (22.4)	7240 (20.13)	
4+ Comorbidities	103,240 (15.53)	26,480 (12.74)	70,960 (16.87)	5800 (16.12)	
**Comorbidities, n (%)**					<0.001
Diabetes	142,620 (21.5)	37,210 (17.9)	97,870 (23.3)	7540 (21.0)	
Hypertension	380,435 (57.3)	116,885 (56.2)	245,100 (58.3)	18,450 (51.3)	
Dyslipidemia	318,030 (47.9)	94,815 (45.6)	209,240 (49.7)	13,975 (38.9)	
Obstructive Sleep Apnea	120,935 (18.2)	40,800 (19.6)	74,330 (17.7)	5805 (16.1)	
Chronic Anemia	33,240 (5.0)	7385 (3.6)	23,495 (5.6)	2360 (6.6)	
Alcohol Abuse	9715 (1.5)	2240 (1.1)	6220 (1.5)	1255 (3.5)	
Mental Disorders	226,910 (34.1)	66,090 (31.8)	146,130 (34.7)	14,690 (40.8)	
Alzheimer’s Disease	2065 (0.3)	300 (0.1)	1590 (0.4)	175 (0.5)	
Parkinsons Disease	7165 (1.1)	1420 (0.7)	5230 (1.2)	515 (1.4)	
Chronic Kidney Disease	58,790 (8.8)	12,975 (6.2)	42,545 (10.1)	3270 (9.1)	
COPD	66,050 (9.9)	15,380 (7.4)	46,675 (11.1)	3995 (11.1)	
Congestive Heart Failure	31,220 (4.7)	6025 (2.9)	23,120 (5.5)	2075 (5.8)	
**Hospital Bed Size, n (%)**					<0.001
Small	210,875 (31.7)	68,410 (32.9)	132,230 (31.4)	10,235 (28.5)	
Medium	177,500 (26.7)	55,630 (26.8)	112,860 (26.8)	9010 (25.1)	
Large	276,170 (41.6)	83,855 (40.3)	175,590 (41.7)	16,725 (46.5)	
**Hospital Location/Teaching, n (%)**					<0.001
Rural	56,695 (8.5)	15,770 (7.6)	37,780 (9.0)	3145 (8.7)	
Urban non-teaching	166,890 (25.1)	51,820 (24.9)	106,690 (25.4)	8380 (23.3)	
Urban teaching	440,960 (66.4)	140,305 (67.5)	276,210 (65.7)	24,445 (68.0)	
**Hospital Region, n (%)**					<0.001
Northeast	101,930 (15.3)	34,765 (16.7)	61,505 (14.6)	5660 (15.7)	
Midwest	184,165 (27.7)	55,635 (26.8)	119,565 (28.4)	8965 (24.9)	
South	249,100 (37.5)	71,320 (34.3)	164,385 (39.1)	13,395 (37.2)	
West	129,350 (19.5)	46,175 (22.2)	75,225 (17.9)	7950 (22.1)	

**Table 3:** Patients’ primary Etiology by Shoulder Replacement Surgery Type.

	Overall (N = 664,545)	ATSA (n = 207,895)	RTSA (n = 420,680)	HA (n = 35,970)
Etiology, n (%)				
Osteoarthritis	433,315 (65.20)	193,910 (93.27)	226,875 (53.93)	12,530 (34.85)
Rotator cuff tear	73,540 (11.07)	1480 (0.71)	71,310 (16.96)	750 (2.09)
Proximal humerus fracture	65,785 (9.90)	1715 (0.83)	55,710 (13.24)	8360 (23.24)
Complication	41,360 (6.22)	5920 (2.85)	25,655 (6.10)	9785 (27.20)
Arthropathies	24,580 (3.70)	680 (0.33)	23,585 (5.61)	315 (0.88)
Avascular necrosis	6860 (1.03)	2395 (1.15)	2405 (0.57)	2060 (5.73)
Deformity	4540 (0.68)	315 (0.15)	3630 (0.86)	595 (1.66)
Inflammatory arthritis	1930 (0.29)	575 (0.28)	1220 (0.29)	135 (0.38)
Malunion/nonunion	1930 (0.29)	80 (0.04)	1470 (0.35)	380 (1.06)
Malignancy	570 (0.09)	30 (0.01)	330 (0.08)	210 (0.58)
Other	8125 (1.22)	445 (0.21)	7165 (1.70)	515 (1.43)

**Table 4:** Summary of complications and hospital outcomes comparing Shoulder Replacement Surgery Type.

	ATSA (n = 207,895)	RTSA (n = 420,680)	HA (n = 35,970)	Odds Ratio (95% CI)
Complications, n (%)	17,395 (8.37)	58,115 (13.81)	6405 (17.81)	1.48 (1.43–1.54)
Urinary Tract Infection	2025 (0.97%)	8100 (1.93%)	900 (2.50%)	1.62 (1.47–1.78)
Respiratory Complications	1885 (0.91%)	7780 (1.85%)	1010 (2.81%)	1.57 (1.42–1.73)
Acute Renal Failure	2040 (0.98%)	9355 (2.22%)	1315 (3.66%)	1.63 (1.49–1.78)
Embolism (DVT, PE)	95 (0.05%)	500 (0.12%)	70 (0.19%)	1.76 (1.19–2.61)
Blood Loss Anemia	12,500 (6.01%)	40,460 (9.62%)	4455 (12.39%)	1.42 (1.37–1.48)
Cardiac Complications	360 (0.17%)	1145 (0.27%)	180 (0.50%)	1.23 (0.98–1.55)
Hospital Outcomes				
Prolonged Length of Stay	17,925 (8.62%)	77,010 (18.31%)	10,840 (30.14%)	1.68 (1.62–1.73)
High-End Hospital Charges	36,650 (17.63%)	119,510 (28.41%)	8805 (24.48%)	1.73 (1.69–1.78)
In-Hospital Mortality	65 (0.03%)	265 (0.06%)	75 (0.21%)	1.10 (0.69–1.73)
Elective surgery	201,695 (97.02)	389,740 (92.65)	28,995 (80.61)	0.72 (0.69–0.75)

**Table 5:** Comparison of hospitalization outcomes between Shoulder Replacement Surgery Type.

	Overall (N = 664,545)	ATSA (n = 207,895)	RTSA (n = 420,680)	HA (n = 35,970)	*p*-Value
Total Charges ($)	64,684 [47,239–90,864]	57,767 [42,525–79,946]	68,523 [50,393–96,464]	59,521 [40,948–90,196]	<0.001
Length of Stay (days)	1 [1,2]	1 [1,2]	1 [1,2]	2 [1–3]	<0.001

## Data Availability

Restrictions apply to the availability of these data. Data were obtained from HCUP and are available [https://hcup-us.ahrq.gov/ (accessed on 1 February 2025).] with the permission of HCUP.

## Abbreviations

The following abbreviations are used in this manuscript:

ATSA	Anatomic Total Shoulder Replacement
HA	Hemiarthroplasty
LOS	Length of Stay
PHF	Proximal Humerus Fracture
RSTA	Reverse Total Shoulder Arthroplasty
